# Maternal antenatal anxiety, postnatal stroking and emotional problems in children: outcomes predicted from pre- and postnatal programming hypotheses

**DOI:** 10.1017/S0033291714001342

**Published:** 2014-07-28

**Authors:** H. Sharp, J. Hill, J. Hellier, A. Pickles

**Affiliations:** 1 Institute of Psychology, Health and Society, University of Liverpool, Liverpool, UK; 2 Institute of Brain, Behaviour and Mental Health, University of Manchester, Manchester, UK; 3 Institute of Psychiatry, King's College London, London, UK

**Keywords:** Internalizing problems, prenatal anxiety, programming effects, sex differences, stroking

## Abstract

**Background:**

Mothers' self-reported stroking of their infants over the first weeks of life modifies the association between prenatal depression and physiological and emotional reactivity at 7 months, consistent with animal studies of the effects of tactile stimulation. We now investigate whether the effects of maternal stroking persist to 2.5 years. Given animal and human evidence for sex differences in the effects of prenatal stress we compare associations in boys and girls.

**Method:**

From a general population sample of 1233 first-time mothers recruited at 20 weeks gestation we drew a random sample of 316 for assessment at 32 weeks, stratified by reported inter-partner psychological abuse, a risk indicator for child development. Of these mothers, 243 reported at 5 and 9 weeks how often they stroked their infants, and completed the Child Behavior Checklist (CBCL) at 2.5 years post-delivery.

**Results:**

There was a significant interaction between prenatal anxiety and maternal stroking in the prediction of CBCL internalizing (*p* = 0.001) and anxious/depressed scores (*p* < 0.001). The effects were stronger in females than males, and the three-way interaction prenatal anxiety × maternal stroking × sex of infant was significant for internalizing symptoms (*p* = 0.003). The interactions arose from an association between prenatal anxiety and internalizing symptoms only in the presence of low maternal stroking.

**Conclusions:**

The findings are consistent with stable epigenetic effects, many sex specific, reported in animal studies. While epigenetic mechanisms may be underlying the associations, it remains to be established whether stroking affects gene expression in humans.

## Introduction

Long-term associations between indices of prenatal stress and child and adolescent emotional and behavioural problems have been reported (O'Connor *et al.* 2002, 2003; van den Bergh *et al.* 2005; Talge *et al.* 2007). Mainly on the basis of animal studies, prenatal (‘fetal programming’) effects are thought to arise from hypothalamic–pituitary–adrenal (HPA) axis dysregulation associated with reduced glucocorticoid receptor (GR) expression (O'Connor *et al.* 2005; Meaney, 2010; Glover, 2011). By contrast, in rodents, postnatal maternal ‘licking and grooming’ (LG) increase GR expression via promoter region demethylation, improving HPA axis regulation, and reducing anxiety behaviours (Meaney & Szyf, 2005; Lemaire *et al.* 2006; Del Cerro *et al.* 2010). In animals the postnatal effects are caused by tactile stimulation so we asked whether, in humans, maternal stroking has the effect that would be predicted from the animal work, i.e. does it reverse prenatal stress effects? Using a self-report measure on two occasions, we asked mothers participating in a longitudinal study, the Wirral Child Health and Development Study, how often they stroked their infants, when they were 5 and 9 weeks old. We found that associations of prenatal depression, with vagal reactivity and temperament at 29 weeks were both modified by maternal stroking over the first weeks of life (Sharp *et al.* 2012). The significant statistical interaction arose because increasing prenatal depression was associated with decreasing vagal reactivity, which is likely to be associated later with poorer emotion regulation, only in the infants of low stroking mothers. Similarly, the association between prenatal depression and increasing negative emotionality, as reported by mothers in a standard measure of temperament, was also seen only in the infants of low stroking mothers.

Programming effects of low fetal growth, and maternal anxiety and depression, may, however, be different in males and females. Some studies of low birth weight (Costello *et al.* 2007; van Lieshout & Boylan, 2010) and prenatal anxiety (van den Bergh *et al.* 2008) have reported associations with adolescent depression in females but not in males. We have reported, from the Wirral Child Health and Development Study, interactions between sex of infant and low birth weight and prenatal anxiety, in the prediction of vagal reactivity at 29 weeks (Tibu *et al.* 2014). Each prenatal risk was associated with increasing vagal reactivity in females, and with decreasing vagal reactivity in males. The plausibility of sex differences is supported by animal studies that find sex differences in physiological, gene expression and behavioural responses to prenatal stress (Weinstock, 2007, 2011; Kajantie & Raikkonen, 2010; Mychasiuk *et al.* 2011; Zohar & Weinstock, 2011). For example, in a number of studies, pregnant rats were exposed to daily random stress during the last gestational week and behaviour tested in the adult offspring. Female, but not male, offspring showed anxiety-related behaviours (Schulz *et al.* 2011), reduced exploration of the open arms of an experimental maze, a marker for increased anxiety (Zagron & Weinstock, 2006), and increased length of immobility in the forced swim test, a model of depression (Frye & Wawrzycki, 2003). Zagron & Weinstock (2006) showed that adrenalectomy of rat mothers eliminated the effect of prenatal stress in female offspring consistent with mediation by effects of maternal corticosteroids on the HPA axis.

We now ask whether there are longer-term effects of stroking up to the age of 2.5 years, whether the patterns of associations remain the same, and whether there is an effect on emotional and behavioural symptoms in young children. This is of importance for four main reasons. First, it is a test of whether the stroking effect endures. If it does it will strengthen the link with animal studies that find long-term effects of LG and it would suggest that tactile stimulation interventions may constitute a viable target for future human early intervention studies. Second, if the pattern of effects is similar at the two time points, with elevated stroking attenuating the associations between prenatal risks and outcomes, it will suggest that the same underlying process is operating at both time points. Third, by this age it is possible to assess symptoms previously found to be associated with prenatal anxiety, in particular anxious/depressed and attentional symptoms, thus addressing whether maternal stroking may be relevant to previously established findings examining programming effects. Fourth, internalizing and externalizing symptoms at 2.5 years are known to be predictive of symptoms in later childhood and adolescence (Bosquet & Egeland, 2006; Kerr *et al.* 2007).

The first hypothesis is that prenatal anxiety will be associated with internalizing and externalizing symptoms at 2.5 years, modified by maternal stroking. Modification will be evidenced in a statistical interaction that reflects that there is an association between prenatal anxiety and symptoms at 2.5 years only in the infants of low stroking mothers. The second hypothesis is that these effects persist after controlling for possible prenatal and postnatal confounders. The third hypothesis is that the effect of stroking will be seen more strongly in females than males as evidenced in a three-way interaction of sex of infant × prenatal anxiety × maternal stroking.

## Method

### Sample

The participants were members of the Wirral Child Health and Development Study, a prospective epidemiological longitudinal study starting in pregnancy with follow-up over several assessment points during infancy up to when the children were 31.37 (s.d. = 2.50) months of age (referred to as 2.5 years). This uses a two-stage stratified design in which a consecutive general population sample (the ‘extensive’ sample) is used to generate a smaller ‘intensive’ sample stratified by psychosocial risk (psychological abuse in the partner relationship) and both are followed in tandem. This enables intensive measurement to be employed efficiently with the stratified subsample, while weighting back to the extensive sample enables general population estimates to be derived. The extensive sample was identified from consecutive first-time mothers who booked for antenatal care at 12 weeks gestation between 12 February 2007 and 29 October 2008. The booking clinic was administered by the Wirral University Teaching Hospital which was the sole provider of universal prenatal care on the Wirral Peninsula, a geographical area bounded on three sides by water. Socio-economic conditions on the Wirral range between the deprived inner-city and affluent suburbs, but with very low numbers from ethnic minorities.

The study was introduced to the women at 12 weeks of pregnancy by clinic midwives who asked for their agreement to be approached by study research midwives when they attended for ultrasound scanning at 20 weeks gestation. Ethical approval for the study was granted by the Cheshire North and West Research Ethics Committee on 27 June 2006 (reference no. 05/Q1506/107). After obtaining written informed consent the study midwives administered questionnaires and an interview in the clinic. Of those approached by study midwives, 68.4% gave consent and completed the measures, yielding an extensive sample of 1233 mothers (mean age = 26.8 years, s.d. = 5.8 years, range 18–51 years) with surviving singleton babies who were available for postnatal follow-up.

Maternal responses to questions about psychological abuse in their current or recent partner relationship (Moffitt *et al.* 1997) were used to generate the stratified intensive sample of mothers for more detailed study. The stratification variable was chosen for its known association with a variety of risk factors for early child development. For this study of prenatal anxiety the stratification variable was effective – the mean State Anxiety scores (Spielberger *et al.* 1983) in the low- *v.* high-risk strata were 31.5 (s.d. = 8.45) *v.* 35.5 (s.d. = 11.17) (Cohen's *d* = 0.40, *p* = 0.001), for comparison of transformed scores. These ‘intensive sample’ mothers completed questionnaire measures of anxiety at a mean of 32.1 (s.d. = 2.0) weeks of pregnancy. We focus here on those with surviving singleton births from this intensive sample who provided repeat assessments of maternal anxiety and maternal stroking and on whom mothers provided ratings of internalizing and externalizing problems at age 2.5 years.

**Fig. 1.**
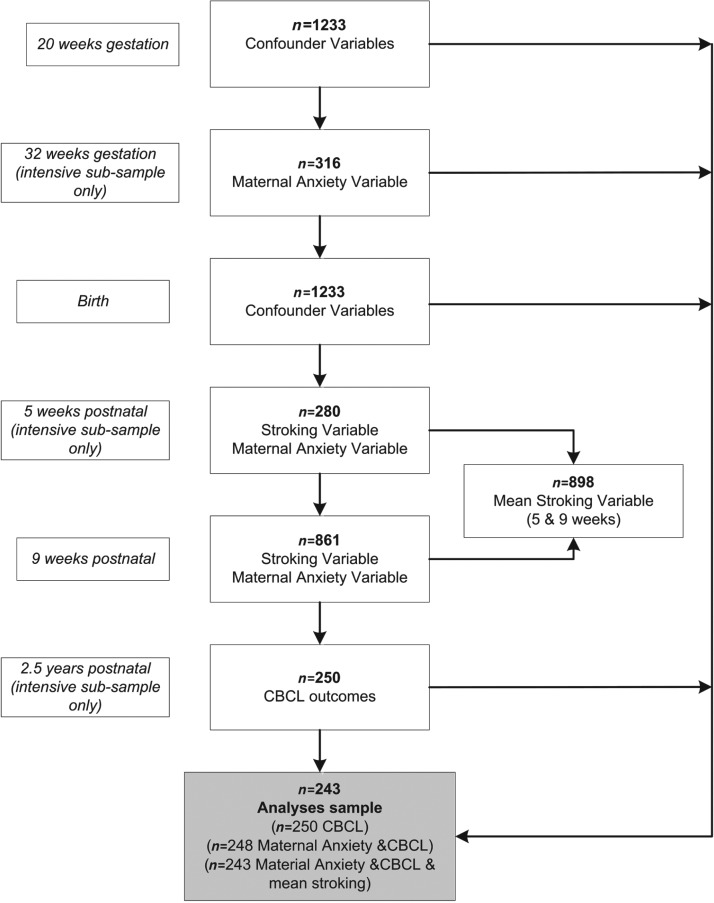
Sample flow. Postnatal maternal anxiety is the mean of scores obtained at 5, 9 and 29 weeks, 14 months and 2.5 years; the 29 weeks and 14 months data points are not shown in the figure. CBCL, Child Behavior Checklist.

Numbers recruited to the extensive and intensive samples and followed up for the measures used in this analysis are shown in Fig. 1. Data were obtained on all 1233 from birth records, and maternal report of stroking on 861 at 9.3 (s.d. = 3.6) weeks (‘9 weeks’). There were 316 mothers recruited to the stratified intensive sample at 32 weeks pregnancy. Of these, 280 provided information on infant stroking when their infants were 5.2 (s.d. = 1.1) weeks old (‘5 weeks’), and 250 mothers provided outcome data for this report when the children were 31.37 (s.d. = 2.50) months old (‘2.5 years’). Mothers providing information at 2.5 years were slightly older than those in the original extensive sample, with a mean age of 27.9 years (s.d. = 6.2 years, range 18–51 years) at recruitment.

In the extensive sample 41.8% were in the most deprived quintile of UK neighbourhoods (Noble *et al.* 2004), consistent with high levels of deprivation in some parts of the Wirral. A total of 48 women in the extensive sample (3.9%) described themselves as other than white British.

### Measures

#### Maternal anxiety and depression

Maternal anxiety was assessed at 32 weeks of pregnancy using the State Anxiety Scale (Spielberger *et al.* 1983), a widely used maternal self-report measure. Postnatal maternal anxiety was assessed using the same measure at 5, 9 and 29 weeks, 14 months and 2.5 years, to control for postnatal effects. The mean across the five assessment points was used as the index of postnatal exposure to maternal anxiety. Maternal depression was assessed by self-report using the Edinburgh Postnatal Depression Scale (Cox *et al.* 1996) at the same time points, and 2.5 years scores were included in analyses to control for possible biasing effects on maternal reports of child symptoms.

#### Maternal stroking

Maternal stroking was assessed by self-report using The Parent–Infant Caregiving Scale (Sharp *et al.* 2012) in which mothers completed four items reporting on how often (1 = never, 2 = rarely, 3 = sometimes, 4 = often, 5 = a lot) they currently stroked their baby's face, back, tummy, arms and legs. The four stroking items clearly assessed a stroking construct as evidenced in high loadings of all of the items on a latent variable in the models. Test–retest reliability over 4 weeks was acceptable (*r* = 0.58). The modification of the effects of prenatal depression by stroking measured in this way was consistent with predictions from the effects of tactile stimulation in rodents. Specificity of the role of stroking was supported by the finding that breast-feeding, which also entails skin-to-skin contact, did not predict vagal reactivity or temperament at 7 months. We used the mean of the measure completed on two separate occasions, at 5 and 9 weeks of age.

#### Child internalizing and externalizing symptoms

Maternal report of child symptoms was assessed at 2.5 years using the preschool Child Behavior Checklist (CBCL), which has been extensively employed in studies of child and adolescent emotional and behavioural disorders (Achenbach & Rescorla, 2000). It has 99 items each scored 0 (not true), 1 (somewhat or sometimes true), and 2 (very true or often true), which are summed to create seven syndrome scales: emotionally reactive, anxious/depressed, somatic complaints, withdrawn, sleep problems, attention problems, and aggressive behaviour. An internalizing grouping total is generated by summing the emotionally reactive, anxious/depressed, somatic complaints and withdrawn scores, and an externalizing total by summing attention problems and aggressive behaviour scores. Standardized T-scores are also generated, and these were used in analyses of externalizing and internalizing symptoms. In line with the authors' recommendations, the syndrome scales, anxious/depressed and attentional problems, were analysed as raw scores.

#### Stratification variable and confounders

Partner psychological abuse was assessed using a 20-item questionnaire covering humiliating, demeaning or threatening utterances in the partner relationship during pregnancy over the previous year (Moffitt *et al.* 1997). Strata were defined using the highest of the partner to participant and participant to partner scores for each family. The sampling fraction for participation in the intensive sample was higher in the high-risk stratum than the low-risk stratum and, as described in the analysis section, stratum weights were used to account for this selection on our results.

Breast-feeding over the first weeks of life was included as a potential confounder variable for maternal stroking since it involves early skin-to-skin contact between mother and infant. Items in the 9 weeks assessment enquired about women's predominant feeding approach with their baby when newborn and at 6 weeks of age. Mothers recorded their responses on a 1–7 scale where the left anchor denoted ‘mostly bottle-fed’ and the right anchor denoted ‘mostly breast-fed’. This response set allowed for variation in the combination of feeding methods adopted at the two time points to be represented in the data. The mean of newborn and 6 weeks breast-feeding scores were used in analyses. Higher scores indicated that the infant was mostly breast-fed.

Demographic and biological risks known to be associated with prenatal stressors and child mental health disorders (Moffitt, 1993; Hill, 2002; Shaw *et al.* 2003; Hutchinson *et al.* 2010; Robinson *et al.* 2011) were included as potential confounders. Variables generated at 20 weeks of pregnancy included mother's age, her cohabiting/marital status, and whether or not she had stayed in education beyond 18 years. Socio-economic status was determined using the revised English Index of Multiple Deprivation (IMD) (Noble *et al.* 2004) based on data collected from the UK Census in 2001. According to this system, postcode areas in England are ranked from most deprived (i.e. IMD of 1) to least deprived (i.e. IMD of 32 482) based on neighbourhood deprivation in seven domains: income, employment, health, education and training, barriers to housing and services, living environment and crime. All mothers were given IMD ranks according to the postcode of the area where they lived and assigned to a quintile based on the UK distribution of deprivation. Variables for drinking alcohol and smoking in pregnancy were derived from information obtained at 20 weeks of gestation from the extensive and again at 32 weeks from the intensive sample. Birth records were used to determine sex of infant, birth weight by gestational age as a measure of fetal growth, and obstetric risk. Obstetric risk was rated using a weighted severity scale developed by a collaboration of American and Danish obstetricians and paediatric neurologists (Beck & Shaw, 2005). The scale has 32 items, each of which has an assigned score in the range 1–5, and the highest-rated item provides the value for analyses. It has been used widely in studies of perinatal complications and later development.

Maternal sensitivity was assessed at 29 weeks with a 15-min standard laboratory-based procedure (NICHD Early Child Care Research Network, 1999). Mothers were asked to play with their infants as they would at home, for 7 min with toys supplied by the mother, and for 8 min with a standard set of toys provided by the experimenter. Maternal sensitivity was rated from video recordings on a global five-point scale, ranging from 1 (not at all characteristic) to 5 (highly characteristic), reflecting mothers' appropriate, supportive, warm responding to infant communications, playful bids or distress. Training on the sensitivity measure was provided by an investigator from the National Institute of Child Health and Human Development (NICHD) Network. Three raters, blind to the other measures, coded sensitivity from video recordings. Each rater achieved good inter-rater reliability for maternal sensitivity on a subset of 30 assessments (intraclass correlation = 0.85–0.91).

### Statistical analysis

The two-phase stratified sample design allows estimates to be reported for the general population from the stratified subsample by the use of inverse sampling probability weights. Weights took account not only of the original stratification but also of the sample attrition that took place up to the assessment at age 2.5 years including mothers' age and years of education, maternal smoking and depression score in pregnancy, and a score of the number of items left incomplete at the initial assessment. Variation in the weights associated with the covariates of each model was removed to improve efficiency.

All analyses were undertaken in Stata Release 11 (StataCorp LP, USA; 2009). Test statistics for weighted means, correlations (using aweights) and regression estimates (using pweights) are based on survey-adjusted Wald tests [*t* tests if single degrees of freedom (df) or *F* tests if multiple df], using the robust ‘sandwich’ estimator of the parameter covariance matrix (Binder, 1983).

To account for the non-normal distribution of CBCL scores, only modest in the case of the internalizing and externalizing T-scores but more substantial for the anxiety/depression and attention subscales, we used a model in which the expected residual variance was made a function of the mean. This model enables us to make sound inference when fitting additive models to the skewed response variables. Because in such models the explained variance changes with the predicted value, they do not provide an overall estimate of the variance explained by the predictors. The model was estimated using an iterative procedure in GLLAMM (Generalised Linear Latent And Mixed Models; S. Rabe-Hesketh, A. Skrondal and A. Pickles, 2005; www.gllamm.org) for estimating heteroscedastic regressions in which the log-standard deviation of the residual error was a function of the predicted mean, a generalization of the mean-variance equality assumed in Poisson regression. The two-way interaction terms were the product of the maternal prenatal anxiety and postnatal stroking scores. The three-way interaction terms were the product of maternal anxiety, stroking and child's sex. The interaction terms were tested in the presence of all lower-level effects by Wald's test. Following Little *et al.* (2006) we centred and residualized the interaction terms to enable lower-order interaction and main effects to remain interpretable as average effects.

For the prediction of internalizing, anxious/depressed, externalizing and attentional symptoms at age 2.5 years we first accounted only for child age and sex, then proceeded to estimates from models that also covaried for the full set of confounders: maternal age, cohabiting/marital status, education, prenatal smoking and alcohol consumption, neighbourhood deprivation, infant birth weight by gestational age and birth complication index, mean postnatal anxiety over five time points, maternal depression at 2.5 years, and mean of newborn and 6 weeks breast-feeding. The effects of the confounders are shown in online Supplementary Table S3. In order to generate data for the figures the same fully adjusted models were run with high and low stroking groups dichotomized at the median in interaction with maternal stroking.

## Results

### Preliminary analyses

Table 1 presents population estimates for the major variables of the analysis. Online Supplementary Table S1 shows associations between continuous variables and online Supplementary Table S2 those with categorical variables. Maternal anxiety in pregnancy showed a small to moderate correlation with the 30-month internalizing and externalizing CBCL T-scores and the anxiety/depression and attention raw scores. Unsurprisingly, prenatal maternal anxiety scores were highly correlated with postnatal anxiety scores. Maternal stroking showed no association with any of the CBCL scores, or indeed with any of the variables that we examined. Breast-feeding was associated with older age at first pregnancy, lower social deprivation and not smoking during pregnancy, and with lower scores on all of the CBCL scales. Not shown in online Supplementary Table S1, there was no association between maternal stroking at 5 and 9 weeks, and observed maternal sensitivity at 29 weeks (*r* = 0.01, *p* = 0.904). Other variables with clear associations with the CBCL scores were maternal postnatal anxiety and depression. More modest associations were also evident for maternal age, neighbourhood deprivation and birth weight by gestational age. There were marked correlations among the CBCL scores themselves.

**Table 1.** Summary of study variables (n = 243)^a^

Assessment period	Measure	
20 weeks gestation	Maternal age, years	26.5 (5.9)
	IMD quintiles, *n* (%)	
	1	95 (39.1)
	2	50 (20.6)
	3	62 (25.5)
	4	16 (6.6)
	5	20 (8.2)
	Marital status, *n* (%)	
	Single	61 (25.2)
	Cohabit	91 (37.4)
	Married	91 (37.4)
	Smoking status, *n* (%)	
	None	152 (62.6)
	Pre-pregnancy	47 (19.3)
	During pregnancy	44 (18.1)
	Higher education: 18+ years, *n* (%)	
	No	104 (42.8)
	Yes	139 (57.2)
	Alcohol use, *n* (%)	
	None/pre-pregnancy	184 (75.7)
	During pregnancy	59 (24.3)
32 weeks gestation	Maternal depression: EPDS	7.5 (4.4)
	Maternal anxiety: state	32.5 (9.0)
Birth	Obstetric risk: risk weighting	2.1 (1.3)
	Birth weight by gestational age, g/week	85.6 (11.30)
	Baby sex, *n* (%)	
	Female	134 (55.0)
	Male	109 (45.0)
Newborn/6 weeks postnatal	Mean breast-feeding score	4.0 (2.4)
5/9 weeks postnatal	Mean stroking score	3.9 (0.6)
2.5 years postnatal	CBCL attention problems, raw score	2.3 (1.9)
	CBCL anxious/depressed, raw score	1.5 (1.7)
	CBCL externalising, T-score	46.7 (9.1)
	CBCL internalising, T-score	45.2 (9.8)
	Maternal depression: EPDS	4.8 (4.9)
	Age of child, months	31.3 (2.4)
5 weeks to 14 months postnatal	Mean postnatal anxiety: state	25.7 (6.2)

Data are given as mean (standard deviation) unless otherwise indicated.

IMD, Index of Multiple Deprivation; EPDS, Edinburgh Postnatal Depression Scale; CBCL, Child Behavior Checklist.

a Summaries are given as weighted averages based on the intensive allocation.

There were no differences between boy and girl infants with respect to levels of exposure to prenatal anxiety or reported postnatal stroking (Table 2). For the CBCL scores at 2.5 years, sex differences in scores were small and non-significant, although the pattern of differences was consistent with previous evidence, with somewhat higher externalizing problems in boys than girls, but higher internalizing problems in girls. In girls, but not in boys, there were significant correlations between prenatal anxiety and scores on each of the four CBCL scales.

**Table 2.** Summaries of maternal stroking at 5/9 weeks, 32 weeks prenatal maternal anxiety and CBCL subscales by sex, and correlation matrices^a^

	Females: mean (s.d.)		Males: mean (s.d.)			
5/9 weeks mean stroking	3.878	0.584	3.877	0.662		
32 weeks gestation anxiety	31.993	8.941	33.163	9.155		
2.5 years internalizing	45.510	9.838	44.817	9.821		
2.5 years anxious/depressed	1.531	1.663	1.376	1.705		
2.5 years externalizing	45.680	8.937	47.877	9.213		
2.5 years attention problems	2.250	1.896	2.466	1.922		
	5/9 weeks mean stroking	32 weeks gestation anxiety	2.5 years internalizing	2.5 years anxious/ depressed	2.5 years externalizing	2.5 years attention problems
Female correlation matrix						
5/9 weeks mean stroking	1.000					
32 weeks gestation anxiety	−0.122	1.000				
2.5 years internalizing	0.064	0.391	1.000			
2.5 years anxious/depressed	−0.053	0.314	0.747	1.000		
2.5 years externalizing	0.063	0.283	0.650	0.522	1.000	
2.5 years attention problems	−0.064	0.336	0.502	0.359	0.785	1.000
Male correlation matrix						
5/9 weeks mean stroking	1.000					
32 weeks gestation anxiety	−0.033	1.000				
2.5 years internalizing	−0.061	0.126	1.000			
2.5 years anxious/depressed	−0.017	0.058	0.762	1.000		
2.5 years externalizing	−0.043	0.121	0.624	0.460	1.000	
2.5 years attention problems	−0.072	0.220	0.430	0.250	0.813	1.000

CBCL, Child Behavior Checklist; s.d., standard deviation.

a Summaries are given as weighted averages.

### Prenatal anxiety, maternal stroking and CBCL internalizing symptoms at 2.5 years

Table 3 shows unadjusted and adjusted regression models, and online Supplementary Table S3 shows the effects of all of the variables used in adjusted analyses. The left-hand columns of Table 3 show the regression models adjusted for child age, with the three-way prenatal anxiety × maternal stroking × child sex interactions, and all two-way interactions and main effects.

**Table 3.** Summary of multivariate regression analyses showing associations between 32 weeks prenatal maternal anxiety, maternal stroking at 5/9 weeks, and CBCL scores at 2.5 years: unadjusted and adjusted results^a^^,^^b^^,^^c^^,^^d^

	Unadjusted						Adjusted					
	Model 1 (pooled)		Model 2 (girls)		Model 3 (boys)		Model 1 (pooled)		Model 2 (girls)		Model 3 (boys)	
	Coeff (s.e.)	*p*	Coeff (s.e.)	*p*	Coeff (s.e.)	*p*	Coeff (s.e.)	*p*	Coeff (s.e.)	*p*	Coeff (s.e.)	*p*
2.5 years CBCL internalizing T-score												
Anxiety	3.997 (0.702)	<0.001	3.989 (0.706)	<0.001	1.930 (2.230)	0.387	3.294 (1.273)	0.010	3.745 (1.709)	0.028	−0.603 (1.003)	0.548
Stroking	0.638 (0.954)	0.503	0.493 (0.835)	0.555	−0.080 (1.220)	0.948	−0.079 (0.983)	0.936	−0.160 (1.151)	0.890	−0.962 (0.967)	0.320
Anxiety × stroking	−1.663 (0.510)	0.001	−1.468 (0.447)	0.001	1.411 (2.212)	0.524	−2.477 (0.766)	0.001	−2.403 (0.897)	0.007	1.215 (0.940)	0.196
Anxiety × stroking × sex	1.914 (1.205)	0.112					3.390 (1.145)	0.003				
Anxiety × sex	−5.761 (2.406)	0.017					−7.320 (2.767)	0.008				
Stroking × sex	−2.854 (4.663)	0.541					−3.238 (4.766)	0.497				
Sex	14.931 (10.601)	0.154					18.488 (10.182)	0.069				
2.5 years CBCL anxious/depressed raw score												
Anxiety	0.551 (0.152)	<0.001	0.569 (0.154)	<0.001	0.017 (0.292)	0.952	0.360 (0.177)	0.042	0.427 (0.188)	0.023	0.066 (0.164)	0.687
Stroking	−0.150 (0.152)	0.321	−0.177 (0.144)	0.219	0.047 (0.256)	0.854	−0.135 (0.152)	0.374	−0.172 (0.149)	0.247	−0.060 (0.107)	0.577
Anxiety × stroking	−0.305 (0.124)	0.014	−0.309 (0.121)	0.010	−0.168 (0.155)	0.277	−0.446 (0.113)	<0.001	−0.403 (0.105)	<0.001	−0.081 (0.117)	0.489
Anxiety × stroking × sex	0.136 (0.176)	0.439					0.309 (0.192)	0.107				
Anxiety × sex	−1.006 (0.505)	0.046					−0.722 (0.447)	0.106				
Stroking × sex	0.591 (0.718)	0.410					0.092 (0.660)	0.889				
Sex	0.506 (1.741)	0.771					0.913 (1.220)	0.454				
2.5 years CBCL externalizing T-score												
Anxiety	2.628 (0.762)	0.001	2.710 (0.652)	<0.001	1.797 (1.111)	0.106	1.067 (0.944)	0.258	2.223 (1.028)	0.031	−2.209 (0.918)	0.016
Stroking	0.223 (1.123)	0.843	0.440 (0.961)	0.647	−0.176 (1.226)	0.886	0.556 (0.838)	0.507	0.283 (0.832)	0.734	0.158 (0.952)	0.869
Anxiety × stroking	−0.444 (0.726)	0.541	−0.639 (0.438)	0.144	0.296 (1.414)	0.834	−0.891 (0.466)	0.056	−0.672 (0.434)	0.122	−0.258 (0.676)	0.703
Anxiety × stroking × sex	−0.247 (1.021)	0.809					0.829 (0.801)	0.300				
Anxiety × sex	−2.903 (2.143)	0.175					−3.729 (1.817)	0.040				
Stroking × sex	−1.141 (4.925)	0.817					−1.253 (4.104)	0.760				
Sex	9.553 (10.810)	0.377					11.098 (9.189)	0.227				
2.5 years CBCL attention problems raw score												
Anxiety	0.649 (0.208)	0.002	0.651 (0.154)	<0.001	0.503 (0.221)	0.023	0.400 (0.149)	0.007	0.568 (0.150)	<0.001	−0.188 (0.183)	0.304
Stroking	−0.177 (0.298)	0.554	−0.182 (0.221)	0.409	−0.100 (0.235)	0.671	−0.198 (0.155)	0.202	−0.264 (0.150)	0.078	0.001 (0.172)	0.995
Anxiety × stroking	−0.060 (0.261)	0.817	−0.139 (0.148)	0.347	0.070 (0.336)	0.834	−0.212 (0.100)	0.034	−0.224 (0.100)	0.026	0.063 (0.294)	0.831
Anxiety × stroking × sex	−0.010 (0.330)	0.977					0.157 (0.250)	0.531				
Anxiety × sex	−0.429 (0.553)	0.437					−0.651 (0.399)	0.103				
Stroking × sex	0.405 (1.141)	0.722					0.889 (0.742)	0.231				
Sex	0.181 (2.302)	0.937					−0.307 (1.563)	0.844				

CBCL, Child Behavior Checklist; Coeff, coefficient; s.e., standard error.

a The table shows coefficient (robust standard error) and significance for the effect of prenatal maternal anxiety and the mean of 5 and 9 weeks maternal stroking, with an interaction of main effects, accounting for conditional weighting in the models. Adjusted models covary for confounding variables. Full results are shown in Supplementary Table S3.

b Models. Model 1: main effects and pooled interaction. Model 2: main effects and interaction – girls. Model 3: main effects and interaction – boys.

c Variables. Variables standardized: 32 weeks gestation anxiety, 5/9 weeks mean stroking, 2.5 years depression, obstetric risk, mean postnatal anxiety. Interactions: replaced by residuals from regression against other model covariates.

d Confounders. Maternal: age, marital status (cohabiting/married/single), higher education, Index of Multiple Deprivation quintiles, smoking status (before pregnancy, during pregnancy, never), alcohol in pregnancy, obstetric risk, 2.5 years depression, mean postnatal anxiety and a mean breast-feeding score. Paediatric: birth weight by gestation age, exact age at 2.5 years data collection, sex.

**Fig. 2.**
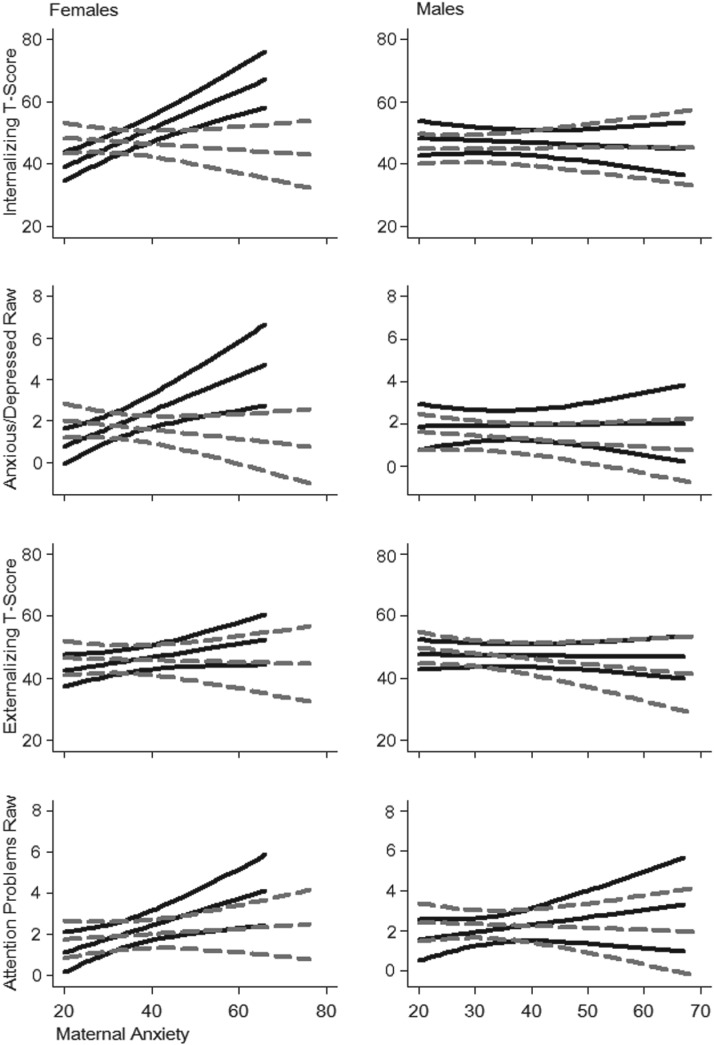
Line graphs displaying high and low stroking with 95% confidence intervals for 2.5 years Child Behavior Checklist subscales plotted against 32 weeks gestation maternal anxiety. Results are presented by sex. Stroking was dichotomized by the median value of mean of stroking scores at 5 and 9 weeks postnatal. High stroking is indicated by the grey dashed line; low stroking by the solid black line. The graphs were generated from fully adjusted models.

Internalizing T-scores at 2.5 years were strongly predicted by prenatal anxiety and by the anxiety × maternal stroking interaction (*p* = 0.001). Although the separate analyses of females and males shown in Table 3 reveal that maternal stroking strongly modified the effect of prenatal anxiety in females, but not in males, the three-way interaction was not significant. However, in the right-hand columns of Table 3, where all the analyses are shown after accounting for confounders^1^†, it can be seen that the two-way interaction prenatal anxiety × stroking, and the three-way interaction prenatal anxiety × maternal stroking × infant sex, predicting internalizing symptoms, were significant (*p* = 0.001 and *p* = 0.003, respectively). In the adjusted regression model, shown in online Supplementary Table S3, breast-feeding was associated with lower internalizing symptoms in girls (*p* = 0.033). After the addition of the prenatal anxiety × breast-feeding interaction, which was non-significant (*p* = 0.598), the prenatal anxiety × stroking interaction and the three-way interaction with infant sex were unchanged (*p* = 0.002 and *p* = 0.008, respectively). The way in which maternal stroking modified the association between prenatal anxiety and internalizing symptoms at 2.5 years is illustrated in Fig. 2, where low and high stroking groups, created using a median split, are contrasted. Dichotomizing stroking gave results consistent with those above for both females and males (interaction *p* < 0.001 and *p* = 0.462, respectively). With increasing prenatal anxiety the daughters of low stroking mothers showed increasing internalizing symptoms, whereas this effect was not seen in girls whose mothers were in the high stroking group, nor was it seen in boys.

Analyses of the CBCL anxiety/depression subscale showed a very similar pattern. Prior to the addition of confounders there was a significant prenatal anxiety × stroking interaction on the subscale score (*p* = 0.014), which was strengthened (*p* < 0.001) on the addition of the confounders to the model. The three-way interaction was of marginal significance (*p* = 0.107), though the model with confounders fitted to boys and girls separately showed a significant anxiety × stroking interaction for girls (*p* < 0.001) but not boys (*p* = 0.489). After the inclusion of the anxiety × breast-feeding interaction (*p* = 0.417), the anxiety × stroking interactions for the overall sample and for girls were unchanged (*p* < 0.001 and *p* = 0.002, respectively). Dichotomizing stroking, the second row of panels in Fig. 2, confirms the similarity of the effects on anxiety/depression to those on internalizing symptoms (interaction *p* = 0.015 for girls and *p* = 0.317 for boys), with a strong association between prenatal anxiety and anxious/depressed symptoms at 2.5 years only in the daughters of low stroking mothers.

### Prenatal anxiety, maternal stroking and CBCL externalizing symptoms at 2.5 years

Prior to the addition of confounders, externalizing T-scores were strongly predicted by prenatal anxiety (*p* = 0.001) but the anxiety × maternal stroking interaction was not significant (*p* = 0.541). There was some evidence for an interaction after the addition of confounders (*p* = 0.056) (Table 3). There was also a main effect of breast-feeding (*p* = 0.009, online Supplementary Table S3), which was largely accounted for by an effect in girls (*p* = 0.001); however, there was not a prenatal anxiety × breast-feeding interaction (*p* = 0.561). The bottom panels of Fig. 2 show the estimated effects, where for girls the interaction with dichotomized stroking was strongly significant (*p* = 0.002) after control for confounders. The findings for the attention/hyperactivity subscale largely mirrored the pattern for the externalizing scores in the simple pooled model in which the prenatal anxiety × stroking interaction was non-significant (*p* = 0.817). However, after the addition of confounders the interaction term was significant for the whole sample (*p* = 0.034), and for girls (*p* = 0.026) but not for boys (*p* = 0.831). There was also a strong main effect of breast-feeding (online Supplementary Table S3, *p* < 0.001), which was evident in both girls (*p* < 0.001) and boys (*p* = 0.001). In addition the prenatal anxiety × breast-feeding interaction was significant in the whole sample (*p* = 0.017) and in boys (*p* = 0.016), but not in girls (*p* = 0.418). After addition of this interaction to the adjusted model the prenatal anxiety × stroking interaction remained significant for the whole sample (*p* = 0.024) and for girls (*p* = 0.045). This pattern of effects with dichotomized stroking, consistent with those for the continuous measure, is shown in the bottom panel of Fig. 2. After control for confounders, the interaction effect with dichotomized stroking was significant for girls (*p* < 0.001) but not for boys (*p* = 0.564).

## Discussion

In this study we tested predictions derived from animal studies of prenatal stress and postnatal tactile stimulation. Such studies show that the effects of prenatal stress are mediated via decreased GR gene expression, and those of postnatal tactile stimulation by increased GR gene expression. In humans, if maternal stroking soon after birth causes increased GR gene expression it should reverse the effect of an index of prenatal stress such as maternal anxiety.

We found that frequency of infant stroking, assessed via maternal self-report at two time points in the early postnatal period, modified associations between prenatal maternal anxiety and internalizing anxious/depressed and attentional symptoms in children aged 2.5 years. The effect was, however, substantially greater in females than in males, and in the case of internalizing symptoms this was supported by a three-way, prenatal anxiety × stroking × infant sex, interaction. As is evident in Fig. 2, high maternal stroking eliminated the association between prenatal anxiety and internalizing and anxious/depressed symptoms, which was, by contrast, substantial in the presence of low stroking. The effects were similar, but less strong for externalizing and attentional problems. These findings are strikingly similar to those we reported previously where an association between prenatal maternal depression and infant negative emotionality at 7 months was evident in the offspring of low stroking, but not high stroking, mothers (Sharp *et al.* 2012). Thus the association between maternal stroking and later adaptation that we showed previously over a period of approximately 5 months is now evident over more than 2 years. It remains to be established whether these longer-term effects are mediated via earlier behaviours.

The strengths of the study include the epidemiological design, and the prospective measurement of anxiety during pregnancy, maternal stroking during early infancy, and behavioural outcomes at 2.5 years. Postnatal measurement of maternal anxiety on five occasions provided a robust test of the specificity of the prenatal effect. This was also examined in the presence of 10 potential confounders, and current maternal depression to control for possible biasing effects of maternal mood. The specificity of maternal stroking was examined by controlling for breast-feeding, which also entails skin-to-skin contact, both as a main effect and in interaction with prenatal anxiety. We used maternal report of stroking as it draws on behaviour that spans contexts in a way that experimental or naturalistic observation of a large community sample could not. We have previously reported support for its construct validity (Sharp *et al.* 2012) and further support will require additional findings consistent with predictions based on the biology of early development, within this and other samples. In the future, demonstrating agreement with observational measures will also be relevant to establishing validity, although such observational measures are generally limited in studies of human development by restricted coverage over place and time, and so cannot straightforwardly be considered as ‘gold standard’. As in the case of temperament research in infancy, in the absence of an agreed ‘gold standard’, self-report and observational measures perform complementary functions and so the further investigation of maternal stroking in infancy is likely to be approached similarly (Rothbart *et al.* 2001). A limitation of the study is that the measures for hypothesis testing were based on maternal report. Main effects may have arisen from shared reporting biases over the time points, although these could not explain systematic differences in associations linked to levels of stroking. Nevertheless it remains to be seen whether analyses based on information from other informants will yield similar results. All of the evidence that we have presented in this and the previous report has indicated that maternal stroking is not associated with other maternal attributes such as levels of depression or anxiety, or observed maternal sensitivity. However, the study has the limitations that we did not have a measure of maternal sensitivity contemporaneous with maternal reports of their stroking, and there may also be other unmeasured maternal characteristics associated with stroking early in life that also influence the outcome studied.

Even though the three-way, sex of infant × prenatal anxiety × maternal stroking, interaction was significant only for internalizing symptoms, sex differences were evident in all of the analyses. In animal studies, sex differences in the effects of prenatal stress on physiological and behavioural indices of offspring anxiety are ubiquitous. As reviewed earlier, this often arises from effects seen in females but not in males. However, this is not always the case, and effects in males, but not in females have been reported (Brunton & Russell, 2010). In humans too, studies have shown effects in females but not males (e.g. van den Bergh *et al.* 2008), and effects in males but not females (e.g. Li *et al.* 2010), while others have found no clear sex differences (O'Connor *et al.* 2002). In both animal and human studies inconsistencies probably arise from variations in types of stressors, their timing, and the way outcomes are assessed. In particular, pregnancy stress may be associated with internalizing symptoms in female offspring but cognitive and externalizing symptoms in males (Glover & Hill, 2012). If this were the case it would provide at least part of the explanation for the predominance of internalizing disorders such as depression in females, and externalizing disorders such as attention deficit/hyperactivity disorder and conduct disorders in males.

While the effect of prenatal stress on anxiety behaviours seen in females appears to be mediated via altered GR gene expression with effects on HPA axis regulation, this may not be the mechanism for cognitive and externalizing symptoms in males (Weinstock, 2011). The findings described here are consistent with this possibility. If, as we have hypothesized, maternal stroking has an effect on GR gene expression, similar to that of tactile stimulation in rodents, and if the link between prenatal stress and anxiety behaviours mediated via altered HPA axis regulation is confined to females, then we would see the effect of stroking only on female offspring. According to this hypothesis we should also see associations between prenatal anxiety and externalizing symptoms that are not modified by maternal stroking, particularly in males. We did not find evidence to support this proposal.

It remains to be seen whether the effects of maternal stroking in infancy described here, and in our previous report, are mediated via GR gene methylation, or methylation of other key genes. The study of patterns of methylation in humans is at a very early stage with limitations arising not only from questions about the relevance of the epigenetics of peripheral cells to central nervous system gene expression, but also lack of clear evidence of the key CpG sites to be examined. Thus while recent findings linking prenatal depression or anxiety, and postnatal environmental exposures to GR gene methylation are promising, they vary substantially in which sites have been examined (Oberlander *et al.* 2008; Hompes *et al.* 2013; Melas *et al.* 2013).

Although the rationale for investigating maternal stroking in this study was the animal evidence for epigenetic effects of tactile stimulation on HPA axis regulation, in humans tactile stimulation has many other effects. Characteristic patterns of prefrontal cortex and limbic activations have been shown in response to stroking with a pleasant stimulus, such as velvet, contrasted with a neutral or unpleasant stimulus, such as sandpaper (Gordon *et al.* 2013). Connectivity between these regions is central to effective emotional and behavioural regulation, and impaired functioning of each region and failures of connectivity have been hypothesized to underpin conditions such as depression and borderline personality disorder (New *et al.* 2007). It is possible that repeated stroking early in development, leading to activations in these regions, may enhance their functions and their connectivity.

Irrespective of the mechanism, we now have increasing evidence from this study that maternal stroking modifies the effects of indices of prenatal stress, such as maternal depression and anxiety, and over substantial periods of time. It remains to be seen whether these effects continue through childhood and beyond, whether they remain confined to internalizing symptoms in females, and whether they are modified by later experiences. If replicated in other samples there will be major implications for our understanding of the interplay between pre- and postnatal influences, and ultimately for the development of treatments and services during pregnancy and early infancy.

## Footnotes

† The notes appear after the main text.

## Note

1 Maternal age, cohabiting/marital status, education, prenatal smoking and alcohol consumption, neighbourhood deprivation, infant birth weight by gestational age and birth complication index, mean postnatal anxiety over five time points, maternal depression at 2.5 years, and mean of newborn and 6 weeks breast-feeding.

## Supplementary material

For supplementary material accompanying this paper, please visit http://dx.doi.org/10.1017/S0033291714001342.
